# Irreducible distal radial fracture due to extensor pollicis longus tendon interposition: a case report

**DOI:** 10.1186/1757-1626-2-6822

**Published:** 2009-06-03

**Authors:** Paraskumar Mohanlal, Sunil Jain

**Affiliations:** Department of Trauma and Orthopaedics, Medway Maritime HospitalMedway, ME7 5NYUK

## Abstract

Various causes have been reported for irreducible fractures of the distal radius. Most of them have been reported in children.

We report a case of irreducible distal radial fracture in an 18-year-old Caucasian male patient. The fracture was not reducible by closed methods and extensor pollicis tendon was found to be interposed during surgical intervention. At one year follow-up, the patient had good radiological evidence of fracture healing and a good functional outcome.

This case report highlights the need for a high index of suspicion to rule out soft tissue interposition in cases of irreducible distal radial fractures and a low threshold for open reduction.

## Introduction

Irreducible distal radial fracture due to tendon interposition is uncommon. Most of them have been reported in children. Various causes have been cited including interposition of extensor tendons [1], flexor tendons [2], periosteal flap [3] and soft tissues [4]. Such injuries appear to be common than is reported in the literature. Recognition is necessary to avoid complications of inadequate reduction.

## Case presentation

**Figure 1.**
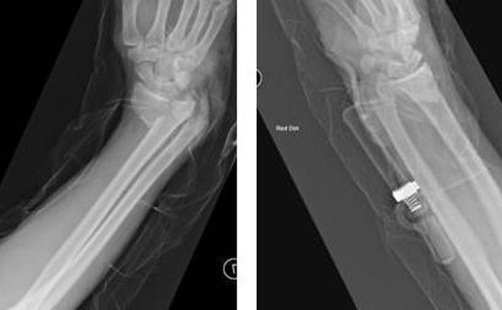
Pre-operative radiographs of the wrist showing displaced fracture of the distal radius and ulna.

An 18-year-old Caucasian male patient presented with a Grade I open distal radial fracture following a road traffic accident. He was hemodynamically stable with no evidence of neurovascular compromise to the limb. Radiographs showed a comminuted, extraarticular volarly displaced, sheer type metaphyseal distal radial fracture associated with fracture of ulnar metaphysis (Figure 1). Patient did not have any other comorbidities and no known allergies to any medication.

The fracture could not be reduced by closed manipulation and hence a volar approach was used for open reduction. However, reduction was still not possible and hence a dorsal approach was used to explore the cause of difficult fracture reduction. The tendon of extensor pollicis longus was found to be interposed in the fracture site. The tendon was released from the fracture site following which the fracture was well reduced. The radial fracture was stabilised with a volar plate and the ulnar fracture with a ‘K’ wire. A prophylactic carpal tunnel decompression was performed.

**Figure 2.**
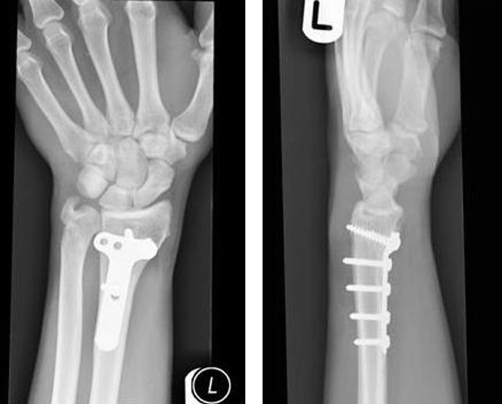
Follow-up radiographs of the same patient showing good radiological evidence of union of both radial and ulnar fractures.

Post-operatively the patient recovered well and at one year follow-up, radiographs showed good fracture healing (Figure 2) and he regained good functional recovery of the arm.

## Discussion

Irreducible fractures of distal radius due to tendon interposition are uncommon injuries in adults. Almost all cases of irreducible distal radial fractures have been reported in children with a growing physeal plate. They have been attributed to interposition of various soft tissues including the flexor and extensor tendons [1-4].

Manoli [3] reported a case of distal radial epiphyseal injury complicated by entrapment of flexor digitorum profundus tendon causing difficulty in reduction. Extensor tendon interposition between the metaphysis and epiphysis of both the ulna and radius was reported by Karlsson et al [1]. Ooi et al [4] described two consecutive cases of Galeazzi-equivalent fracture in children associated with tendon interposition at the fracture site and reported good functional outcome after open reduction and internal fixation. Periosteal flap causing difficult fracture reduction in Salter Harris type II distal radial injury was reported by Lesko et al [3].

In our case the patient had attained skeletal maturity and sustained this fracture due to high velocity injury. The mechanism of injury due to fall from a motor bike, fracture of both distal radius and ulna, associated soft tissue disruption, and a sheer type fracture configuration of the radius would have caused rupture of the extensor retinaculum and consequent entrapment of the extensor pollicis longus tendon at the fracture site. This was identified by dorsal approach during open reduction. Release of the tendon resulted in satisfactory fracture reduction and follow-up radiographs showed good radiological evidence of union.

## Conclusion

This case report highlights the need for a high index of suspicion to rule out soft tissue interposition in cases of irreducible distal radial fractures and a low threshold for open reduction.

## Abbreviations

None.
